# Hepatitis C virus genotype/subtype distribution and evolution among Chinese blood donors: Revealing recent viral expansion

**DOI:** 10.1371/journal.pone.0235612

**Published:** 2020-07-10

**Authors:** Yu Zhang, Zhan Gao, Shaoli Wang, Jing Liu, Ness Paul, Tao He, Cunxu Liu, Hongbin Zhang, Yunlai Lv, Ru’an Cao, Wei Mao, Jianhua Wan, Hongli Ma, Mei Huang, Yu Liu, Jingxing Wang, Pu Liao, Peibin Zeng, Miao He, Hua Shan

**Affiliations:** 1 The Fourth Affiliated Hospital Zhejiang University School of Medicine, Yiwu, China; 2 Institute of Blood Transfusion, Chinese Academy of Medical Sciences, Chengdu, China; 3 Sichuan Blood Safety and Blood Substitute International Science and Technology Cooperation Base, Chengdu, China; 4 The Johns Hopkins Medical Institutions, Baltimore, MD, United States of America; 5 Chongqing Blood Center, Chongqing, China; 6 Guangxi Blood Center, Liuzhou, Guangxi, China; 7 Urumqi Blood Center, Urumqi, Xinjiang, China; 8 Luoyang Blood Center, Luoyang, Henan, China; 9 Mianyang Blood Center, Mianyang, Sichuan, China; 10 The People’s Hospital of Chongqing, Chongqing, China; 11 West China School of Public Health and West China Fourth Hospital, Sichuan University, Chengdu, Sichuan, China; 12 Stanford University, Stanford, CA, United States of America; University of Cincinnati College of Medicine, UNITED STATES

## Abstract

Hepatitis C virus (HCV) genotype (GT) distribution in China shows significant geographical and demographic difference. As a routinely tested virus in Chinese blood bank systems, rare molecular epidemiology research in blood donors is reported. Our purpose is to investigate the HCV GT/subtypes distribution, phylogenetic analysis and population genetics in Chinese blood donors. Anti-HCV screen positive samples and donor demographics were collected. HCV Core and E1 gene fragments were amplified by RT-PCR, followed by sequencing and phylogenetic analysis to determine HCV GTs/subtypes using MEGA 7.0. The population genetics were performed using Arlequin v3.0 and Beast v1.10.4. SPSS Statistics 17.0 software was used to analyze the correlation between HCV GTs/subtypes distribution and demographic characteristics. 419 and 293 samples based on Core and E1 gene respectively were successfully amplified. HCV la, lb, 2a, 3a, 3b, 6a, 6e and 6n were found, and the corresponding proportions were 0.66% (3/455), 58.68% (267/455), 17.80% (81/455) and 5.05% (23/455), 3.52% (16/455), 12.31% (56/455), 0.88% (4/455) and 0.66% (3/455). Samples from Guangxi showed the most abundant genetic diversity with 8 subtypes were found. The number of haplotypes in HCV-1b is higher than 2a and 6a. The negative Tajima's *D* and Fu's *Fs* values of HCV-1b, 2a and 6a suggested the population expansion of those HCV subtypes. The distribution of HCV GT showed significant statistical difference by age and ethnicity. **Conclusion:** An abundance of HCV genetic diversity was found in Chinese blood donors with mainly 1b and then 2a subtype. There were significant geographical and demographic differences in HCV GTs/subtypes among Chinese blood donors. HCV subtype 1b has stronger viability and HCV subtype 6a has experienced significant expansion.

## Background

Hepatitis C Virus (HCV) is a single positive strand RNA virus with envelope belonging to the Flaviviridae family. It can cause both acute and chronic hepatitis, ranging in severity from a mild illness lasting a few weeks to a serious, lifelong illness including Liver cirrhosis, liver cancer and liver failure [1]. WHO has estimated 71 million people have chronic hepatitis C infection. In China, the proportion of new HCV cases is 0.06 ‰ per year, which is even higher in some regions such as Fujian, reaching 6.01% in 2010 [2].

Hepatitis C is mainly transmitted via blood transfusion and plasma derivatives usage before Blood Donation Law initiated in 1998. With the Chinese government banned the paid blood donation, the prevalence of HCV remains stable after a significant reduction in blood donors. Nowadays, HCV infections caused by sexual transmission has been increasing continuously in recent years. [3,4]. While the initiation of HCV infection is concealed, it still remains as a huge threats to Chinese blood safety.

With high variable genome, the diversity of HCV nucleic acid sequence is as high as 30%, and according to which, HCV is divided into 7 GTs and more than 90 subtypes [5]. HCV GT distribution shows geographical differences: HCV-1, HCV-2 and HCV-3 are widely distributed globally; The major HCV subtypes include 1b, 2a, 3a, 3b and 6a in China, of which subtype 1b accounts for more than 70%, followed by 2a [5]; 1b and 2a are more common in north and west China, while 3a, 3b and 6a are more popular in southwest and south China. Besides, recently, some novel group of HCV variants has been found in China [6].

The distribution of HCV GT is diverse in different subgroups such as blood donors, intravenous drug users (IDU) and dialysis patients. In the group of blood donors and blood recipients, the popular subtypes were HCV-1b and 2a [7] but had shown a decrease in some regions with HCV-3 and 6 on the rise [7]. HCV-2a was also found in people who have high-risk sexual behaviors or unsafe medical injection [8].

Characteristics of HCV-infected people among Chinese blood donors were reported [9–11]. In recent years, with the increasing population mobility and the diversified transmission modes, the distribution of HCV GT in blood donors of China is undergoing significant changes [12]. An update on HCV GT distribution among blood donors is needed to re-evaluate the blood screening strategy in blood screening to avoid unnecessary cost and waste of collected units due to false positive results [13]. In this study, plasma samples from anti-HCV positive blood donations were collected from five Chinese blood centers (Luoyang, Chongqing, Mianyang, Urumqi and Guangxi) in three consecutive years (2012–2014). Our aims were: 1) To understand HCV GT and subtype distribution in Chinese blood donors; 2) to investigate the expanding trends of HCV GTs/subtypes using HCV phylogenetic and population dynamics analysis; 3) To provide molecular epidemiology supporting data to improve nucleotide acid test (NAT) assay development based on HCV genome diversity, and to inform public health policy making regarding HCV prevention and control.

## Materials and methods

All methods were carried out in accordance with relevant guidelines and regulations, and The Ethical Review Committee (IORG0006536) of Chinese Academy of Medical Sciences/Peking Union Medical College approved the study and the ethics approval number was X101222002. Before sampling, the voluntary blood donors have signed an informed consent form.

### Study population

802 anti-HCV positive plasma samples were collected from 5 Chinese blood centers including: Chongqing Blood Center, Chongqing; Urumqi Blood Center, Urumqi, Xinjiang; Luoyang Blood Center, Luoyang, Henan; Mianyang Red Cross Blood Center, Mianyang, Sichuan; Guangxi Blood Center, Liuzhou, Guangxi. Samples were collected with EDTA anticoagulation tube, stored at -20°C, and then transported to the Institute of Blood Transfusion, Chinese Academy of Medical Sciences through the cold chain.

The study population was divided into different age groups: 18–20, 21–30, 31–40, 41–50, >50 years of age; Ethnicity group: Han, Hui, Uygur, Zhuang; Occupation groups: student, commercial, worker, peasant, working at home, other; Educational levels: ≤primary school, middle or high school, >high school, other, missing.

### HCV RNA extraction and reverse-transcription polymerase chain reaction (RT-PCR)

The required 8 primers including outer and inner amplification were designed after reference [14]. At present, three most commonly used HCV coding regions to genotyping are Core, E1 and NS5B [15–17], which represents high accuracy and sensitivity, however, sometimes different HCV subtypes can be obtained with the above three regions for the same HCV genome, suggesting the presence of mixed infection or gene recombination [18,19]. In our study, coding regions of Core and E1 were separately used to reconstruct independent HCV GTs/subtypes phylogenetic trees in order to make more accurate genotyping, and the final amplified HCV products based on Core and E1 gene fragments were 401bp and 592bp, respectively.

HCV RNA was extracted from 140μL of each plasma sample by the principle of Membrane adsorption using the QIAmp Viral RNA Mini kit (QIAGEN, German) following the instructions strictly, and then it was reverse transcribed into DNA fragment with the help of antisense outer primers of Core and E1 using the Transcriptor First Strand cDNA Synthesis Kit (Roche, Switzerland). The nested PCR based on Core and E1 gene fragment was performed using 5μL of the RT-PCR product with 2×TSINGKE MASTER MIX (TSINGKE Biotech Co Ltd, Chengdu, China), and primers as specified previously [20]. The final PCR products were confirmed by 2% agarose gel electrophoresis and visualization with ethidium bromide staining, and amplified products were sent to TSINGKE Biotech Co Ltd (Chengdu, China) for purifying and sequencing with the sense and antisense inner primers.

### HCV genotyping, phylogenetic and evolutionary analysis

All the sequences of the Core and E1 regions were checked and spliced using Mega 7.0, and the spliced HCV sequences were aligned with HCV reference sequences (S1 Table). Phylogenetic analysis was performed using the neighbor-joining and the Kimura two-parameter method to get the accurate HCV genotyping. Node supports were evaluated with 1000 bootstrap replicates. HCV GT/subtype was identified when it was included in a monophyletic cluster with classified reference strains.

Arlequin v3.0 was used to perform population genetics data analysis including calculating the haplotype frequencies, estimating the past population expansion of species by Tajima's *D* and Fu' s *Fs* value, and if the values are negative and P <0.05 or 0.01, representing the population is considered to have undergone expansion. BEAST v1.10.4 was used to get the Bayesian skyline plot (BSP) to analyze growth history of different HCV subtypes. Uniform prior was selected and the evolutionary rates of Core and E1 fragment for each dominant subtypes were shown in S2 Table.

### Statistical analysis

Statistical data analyses were performed with SPSS Statistics 17.0. The Pearson Chi-Square test was used to compare the ratio of risk factors between HCV GT/subtypes and demographic characteristics (age, gender, ethnic, occupation, educational level), and P value < 0.05 was considered to be statistically significant.

## Results

### HCV genotyping and phylogenetic analysis

**Fig 1:**
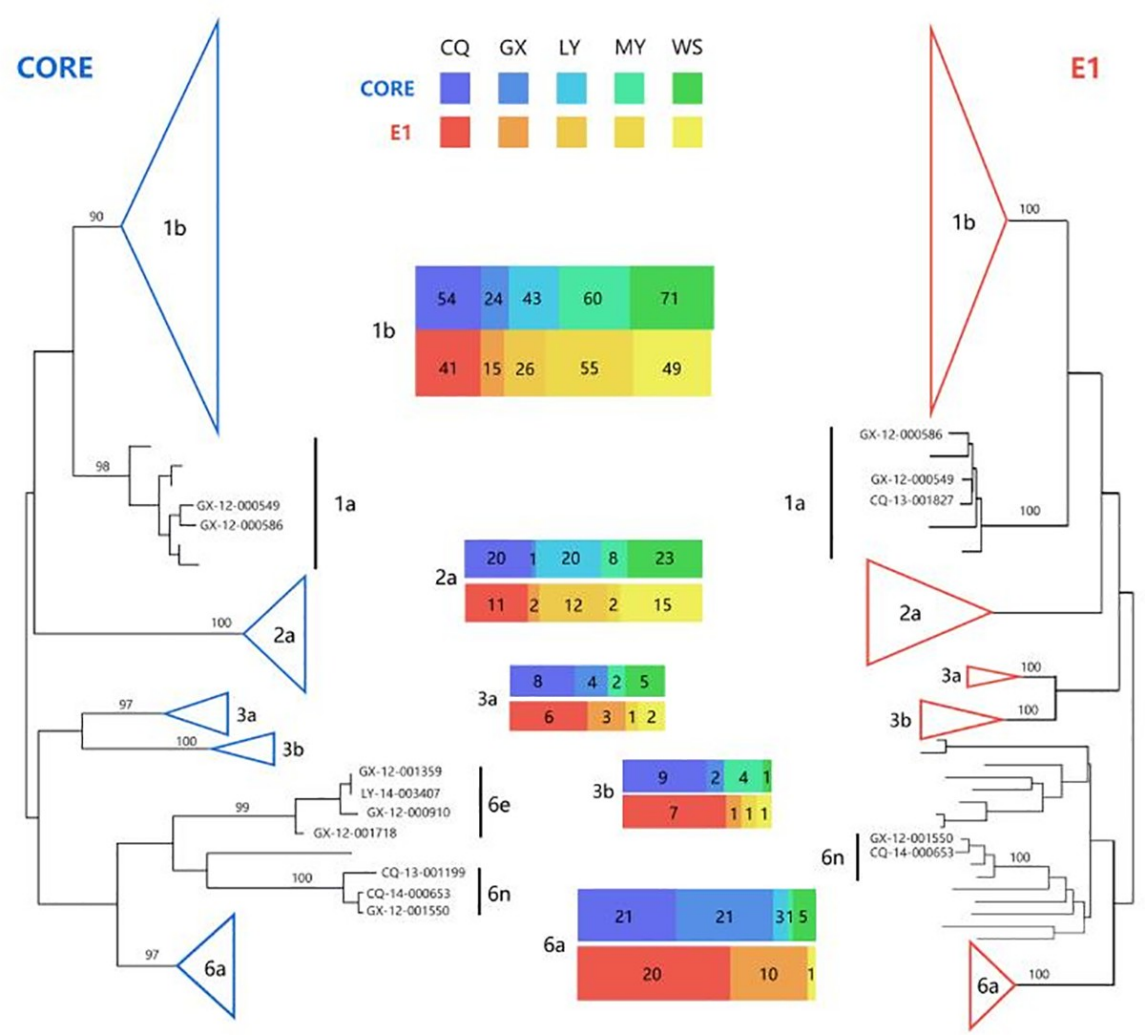
Phylogenetic tree constructed using HCV Core and E1 gene sequences from blood donors in China. Phylogenetic analysis was performed using the neighbor-joining and the Kimura two-parameter method to get the accurate HCV genotyping, and the node supports were evaluated with 1000 bootstrap replicates. The colored triangles and vertical lines represent different HCV subtypes. The upper bar refers to the Core region and the lower bar refers to the E1 region. The colored rectangular area represents the number of HCV subtypes in five regions including Chongqing (CQ), Guangxi (GX), Luoyang (LY), Mianyang (MY) and Urumqi (WS).

Among the 802 anti-HCV positive plasma samples, 419 and 293 were successfully amplified based on Core gene fragment and E1 gene fragment correspondingly. The determined genotype among samples that were successfully amplified by both Core and E1 fragment were consistent, and 36 subtypes, that were successfully amplified by E1 fragment, were not amplified by Core fragment. Based on the above, 455 of 802 samples based on Core and (or) E1 fragment were successfully amplified. The successfully amplified samples were then sequenced and used to construct phylogenetic trees (Fig 1). In detail, the predominant HCV subtype was 1b (58.68%, 267/455), followed by subtype 2a (17.80%, 81/455). Other HCV subtypes including la, 3a, 3b, 6a, 6e and 6n were also found among these blood donors, and the proportion of the above subtypes were 0.66% (3/455), 5.05%(23/455), 3.52% (16/455), 12.31% (56/455), 0.88% (4/455) and 0.66% (3/455), respectively.

The distribution of HCV GT among blood donors in China showed significant geographical difference (P <0.05) (S3 Table). The predominant HCV subtypes in different regions were as followed: 1b (59.72%, 43/72) and 2a (34.72%, 25/72) in Luoyang, 1b (46.21%, 61/132) and 6a (18.94%, 25/132) in Chongqing, 1b (80.77%, 63/78) and 2a (10.26%, 8/78) in Mianyang, 1b (67.57%, 75/111) and 2a (22.52%, 25/111) in Urumqi, and 1b (41.94, 26/62) and 6a (35.48%, 22/62) in Guangxi.

### HCV GT and demographic characteristics

Table 1 showed the association between some HCV subtypes (1b, 2a, 3a, 3b and 6a) and gender, age, ethnicity, education status and occupation. There were significant differences between HCV GT and age and ethnicity (*P* < 0.05), while no statistical differences were observed between HCV GT and gender, occupation and education level (*P* value > 0.05). In the age groups of 21–30, 41–50 and >50, HCV subtypes 1b and 2a accounted for a higher proportion, while in the age groups of ≤20 and 31–40, lb and 6a were more common. In Han population, HCV lb and 2a were the prevalent subtypes, accounting for 72.6% (312/430) of the whole ethnic groups, while HCV 6a was more common in Zhuang population.

**Table 1:** The association between HCV genotypes/subtypes and demographic characteristics.

HCV GTs Demographic characteristics		HCV-1b (n = 267)	HCV-2a (n = 81)	HCV-3a (n = 23)	HCV-3b (n = 16)	HCV-6a (n = 56)	P*
Gender	Male/female	162/105	46/35	13/10	10/6	29/27	>0.05
Age	≤20	3 (**75**)	0	0	0	1 (**25**)	<0.05
	21–30	89 (**63.12**)	32 (**22.70**)	9	3	9	
	31–40	36 (**46.15**)	4	8	6	21 (**26.92**)	
	41–50	106 (**61.99**)	26 (**15.20**)	4	6	23	
	>50	33 (**57.89**)	19 (**33.33**)	2	1	2	
Ethnicity	Han	239 (**60.97**)	73 (**18.62**)	19	13	42	<0.05
	Hui	3 (**60.00**)	1	0	1	0	
	Uygur	14 (**70.00**)	3	3	0	0	
	Zhuang	3 (**14.29**)	1	1	1	13 (**61.90**)	
Education status	>High school	74	26	7	2	14	>0.05
	Middle or high school	159	45	11	14	29	
	≤Primary school	4	3	0	0	4	
Occupation	Student	36	10	2	0	4	>0.05
	Commercial	36	9	3	2	6	
	Worker	24	13	2	2	8	
	Peasant	51	14	2	1	9	
	Working at home	64	21	7	6	15	

* using method of Fisher's exact test

The values in brackets mean the higher proportion of each group.

### HCV population dynamics

The dominant subtypes were 1b, 2a, and 6a. The subtypes determined by Core gene were 251, 72 and 51, respectively and the corresponding haplotypes of Core 1b, 2a and 6a were 178, 55 and 44; The sequences of 1b, 2a, and 6a determined by E1 gene were 186, 42 and 31, respectively and the corresponding haplotypes of E1 1b, 2a and 6a were 183, 42 and 31 (Table 2). The neutral test values of Tajima's *D* and Fu's *Fs* of each HCV subtypes were negative, the *P* value corresponding to Fu's *Fs* of each subtype was < 0.01, and the *P* value corresponding to Tajima's *D* of subtype 1b was < 0.05; The Tajima's *D* and Fu's *Fs* values of HCV 6a were both negative and *P* value is less than 0.01 (Table 2).

**Table 2:** HCV population genetic characteristics in China.

HCV GTs	n	Haplotypes	Tajima's *D*	*P*	Fu's *Fs*	*P*
Core-1b	251	178	-1.48031	0.025*	-24.06017	0.002**
Core-2a	72	55	-0.83021	0.204	-24.20050	0.000**
Core-6a	51	44	-1. 80712	0.009**	-25. 44373	0.000**
E1-1b	186	183	-0.87571	0.213	-23.75550	0.005**
E1-2a	42	42	-0.10326	0.549	-14.12353	0.001**
E1-6a	31	31	-0.88458	0.186	-11.69440	0.003**

*P < 0. 05 significant population expansion

**P < 0. 01 highly significant population expansion.

**Fig 2:**
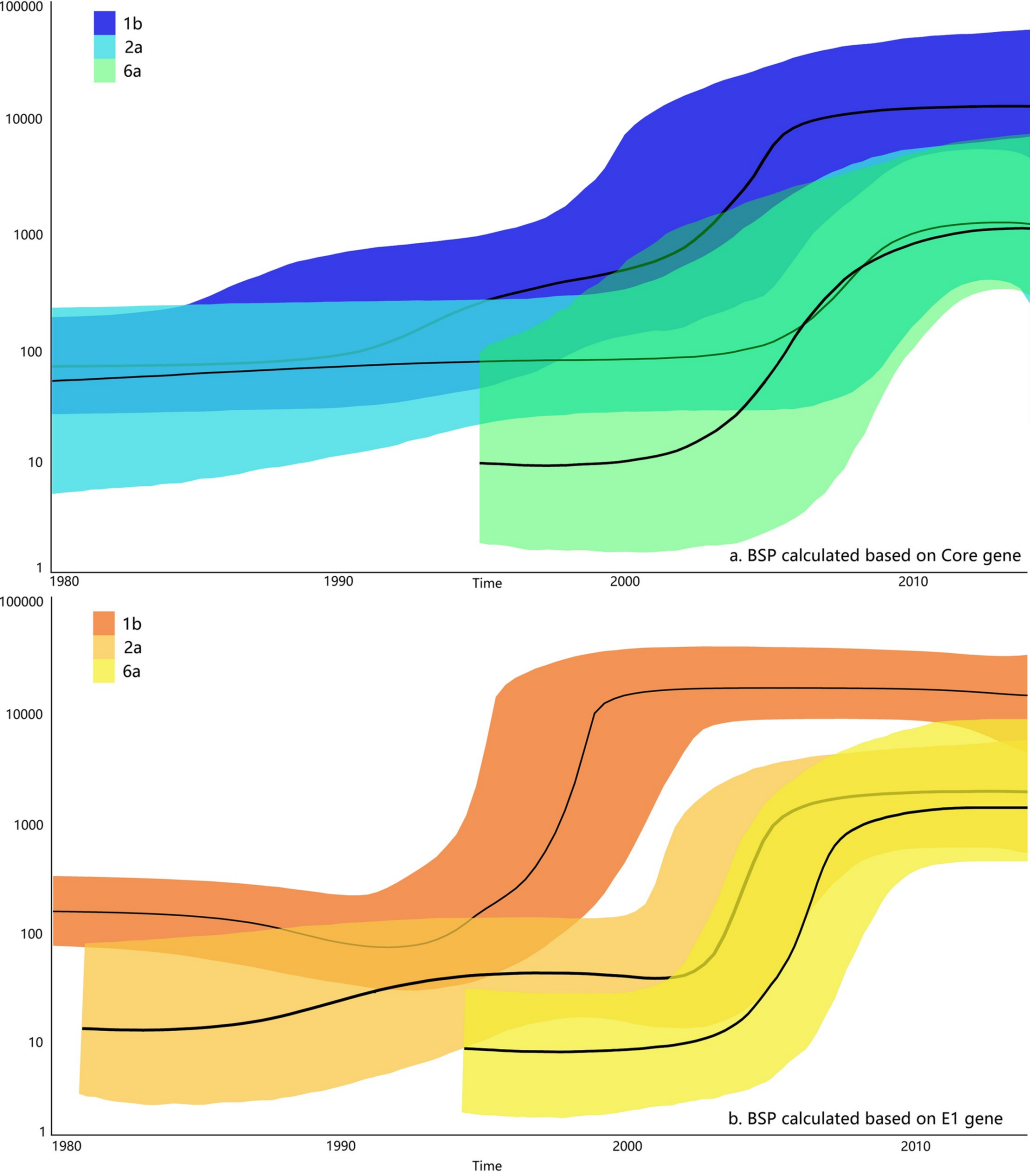
Population genetic characteristics of HCV with different genotypes among blood donors in China. The solid black part is the median of the estimated changes in genetic diversity. Other colored parts is the 95% confidence interval. The horizontal axis is the year. The vertical axis is Neτ (the effective population size of the experimental sample). a: The BSP for HCV 1b(blue), 2a(light blue) and 6a(green) based Core gene. b: The BSP for HCV 1b(orange), 2a(light orange) and 6a(yellow) based E1 gene.

The spatio-temporal changes estimated in the HCV population size were illustrated as results of BSP shown in Fig 2A and 2B. For HCV-1b the Core and E1 genes showed mutually rapid population growth between 1992 and 1998. While the Core gene for HCV-1b showed another period of continuous population growth until 2005. As for HCV-2a, both Core and E1, the genes mutually showed population growth after 2000. HCV-6a was estimated as a new member of the blood donor population that appeared around 1995 and the sudden population expansion occurred between 2002 and 2010, based on the calculation of BSP of the Core and E1 genes.

## Discussion

### Regional differences

In this study, HCV GT/subtype distribution in groups of blood donors showed significant geographical variations that are different from findings in previous publications.

In Guangxi, two studies performed in 1990s showed that the prevalent GTs were 1b and 1a, 3b and 1b [21,22], respectively. However, our study together with reports in recent years, suggest that HCV 1b, 6a has become the dominant subtype over time in Guangxi, which is consistent with a meta-analysis reported [12]. In addition, we also found that the proportion of HCV-6a increased compared with other previous reports in Guangxi while the proportion of HCV-2a were decreasing [21–26]. In early years of 1990s, HCV-2b has been detected in Guangxi [21,22], and since then, this subtype hasn’t never been found in later studies including this one. HCV-6n was detected which was never been reported before in Guangxi. Probably due to the special geographical environment of Guangxi and its proximity to Hong Kong, Macao, Vietnam, Thailand and other countries and regions, such as the HCV transmission of rare genotypes in these countries and regions; coupled with the wide communication leading to HCV genotype in Guangxi extremely rich and complicated.

In Mianyang (Sichuan province), Luoyang (Henan province) and Urumqi (Xinjiang province), the prevalent genotypes were both HCV 1b and 2a. According to the previous reports, the major two HCV subtypes in Sichuan were 1b and 3b or 3a [27–30]. In our study, however, HCV-1b and 2a became the predominant subtypes in Mianyang with the prevalence rate of 1b up to 80%, and the prevalence of 2a was significantly higher than 3a and 3b. In Urumqi, the prevalence of two major subtypes was consistent with three previous reports [31–33]. With limited previous studies about HCV genotypes, the changing trend of each HCV GT in the above two regions can’t be easily found over time. In Luoyang, the prevalent HCV subtypes were consistent with most of previous studies reported, while HCV-2a exhibits a trend of slight increase.

In Chongqing, the distribution of predominant HCV subtypes showed high gene abundance that HCV-1b and 2a [34–36], 1b and 3b [37,38], 2a and 3a [39] have ever been reported as the major HCV subtypes. Comparing with a 2003–2004 study performed in blood donors of Chongqing [39], the proportion of HCV 6a has increased significantly, which was consistent with pooled results of a meta-analysis [12].

The distribution of HCV subtypes had undergone some changes over time in some places of China, just as what we have found in Guangxi, Chongqing and Mianyang. The emergence of new HCV subtypes and changing trends of HCV GTs may be ascribed to: 1) Increasing population mobility: With the increase of domestic or international trade and communication, the interaction between people will naturally increase, which may lead to the spread of HCV; 2) The changing transmission routes: In recent years, there was an increase in HCV IDUs, possibly due to common syringes contaminated by blood, which may represent an increasing of HCV subtypes 3 and 6 [8]. In our study, the increasing proportion of HCV-6a in Guangxi and Chongqing may be a consequence of IDU related spreading in these two areas [25,38]; 3) The improved sensitivity and specificity of testing methods and the sample size. Most notably, a multi-center large sample research still may need to be performed to reveal more meaningful results showing HCV changing trends.

### Demographic association

Reports about the association between HCV GTs and host demographics in China were inconsistent [8,40]. Subtype 1b was higher in male than in female in some reports [41]. The possible reason may be that gender difference exhibits different immune response against HCV GTs and the life style of male population is complex and thus the chances of infecting HCV are relatively high. Previous studies found that in the older group (≥ 40), HCV-1b and 2a were the predominant subtypes, while in the younger group, the proportion of HCV-6 and 3 showed an increasing trend with drug use or high-risk sexual behavior by young people [42,43].

In our study, significant statistical result was found between HCV GTs and age. HCV subtypes 1b and 2a are more common in the age groups of 21–30 and >41, while lb and 6a are more common in the younger groups of 18–20 and 31–40, which was consistent with early reports, while higher proportion of 1b and 2a was found for the group of 21-30.We speculate that it may take years from most HCV infected individuals to develop clinical symptoms [44], thus HCV lb and 2a still remained the main subtypes, and in addition, sampling deviation should also be taken into account.

In Han population, lb and 2a accounted for 72.6% of the whole ethnic groups, while HCV 6a is more common in Zhuang population, and the possible reasons are: 1) The lifestyle of different nationalities are diverse, and HCV transmission modes are also diverse [45]; 2) different ethnicities show different immune responses to HCV infection with different subtypes [46]. In addition, there was no significant association between HCV GTs and occupation, educational level in our study, which was similar to the previous reports [11,47]. The sample size in this study is large and representative; however, the exact mechanism of the relationship between HCV GT distribution and demographic characteristics needs more investigation.

### Population dynamics

BSP analyses were performed using the sequences determined in this study and it showed that HCV 1b, 2a and 6a have experienced different rates of population expansion at different times in the past.

#### BSP for HCV 1b

As shown in BSP 1b based on Core and E1 gene fragment, that they concurrently highlighted the growth in the HCV-infected population size from 1992 to 1998, consistent with a period when an official plasma donation campaign and inadequate donor screening protocol resulted in contaminated blood supply and infected blood donors. This 1990s campaign in Henan province resulted in more than 500,000 blood donors infected with HCV [48–51]. To eliminate this risk, the central government of China banned and outlawed paid blood donation in 1998 [49], and this corresponding change was well reflected in our Core and E1 1b BSP that since 1998, this population expansion tended to be stable. In addition, we found that Core-1b population still through transient expansion after 2000 (2000–2005). We do not deny different sample sizes, evolution rate estimation, parametric model and other aspects resulting this divergence, but a more reasonable explanation, we think, may be that although the enactment of the blood donation law and corresponding knowledge publicity of blood donation reduced the HCV spreading to a certain extent, after all, the infected population is large, and together with population mobility and other factors, allowing wide HCV dissemination. However, in this study, based on BSP results, we believe that HCV 1b is the specific HCV subtype who was the prime culprit of the huge disaster to Chinese blood safety. After the sudden expansion of 1b population, HCV 1b has remains as the main HCV subtype distributed in China.

#### BSP for HCV 2a

Previous reports have showed that unsafe medical practices have a certain relationship with HCV-2a [52]. Since 2004, HCV outbreaks in Anhui, Hubei, and Fujian due to the reuse of unsterilized or inappropriately sterilized needles have been reported [53,54], consistent with the rapid expansion of HCV 2a population in 2005 with BSP. Our BSP based on Core and E1 gene showed that HCV-2a population expanded from 1990 to 2010 at different rates, and according to the above cases of unsafe medical practices, we speculate that HCV transmission occurred from time to time with improper medical behaviors especially in some poor or less-developed regions, resulting in the expansion of this population. In addition, since 2a is the prevalent HCV GT in HCV infection transmitted by blood transfusion [11], and HCV infection caused by blood transfusion or blood products may cause expansion of HCV-2a population [55]. These findings emphasize the importance of tracking HCV outbreaks caused by improper needle reuse or unsterilized injections in order to reduce or eliminate further HCV infection.

#### BSP for HCV 6a

Previous studies have reported that HCV-6 was more frequent among IDU patients [56–58]. Before 2000, transfusion of contaminated blood or blood products was the main route of HCV transmission in China, however, some risk factors such as using illegal intravenous drugs, unsafe medical practices and risky behaviors of homosexual men have become the main HCV transmission routes now [59], which greatly promoted the spread of HCV and also built the best conditions for rapid HCV molecular evolution [60,61]. Our study showed that HCV-6a population undergone population expansion in 2000–2010, which is also corresponding with the above reports [24–26] that the proportion of HCV-6a in Chongqing and Guangxi increased. This may represent that HCV-6a had a tendency of expanding to the general population including blood donor. In addition, HCV-6a expanded relatively more recently compared with the HCV-1b populations.

This study has its limitation in that samples successfully amplified based on the E1 were less than Core, resulting from degradation of nucleic acid with E1 gene fragments being amplified at a later time after Core and this experiment was carried out among blood donors.

In summary, the haplotype diversity in the population is an indicator to measure the population genetic diversity, and the more haplotype the population has, the richer genetic diversity is and the stronger the adaptability of the species to the environment will be. In this study, the number of haplotypes in each HCV subtypes is large, and subtype 1b is higher than 2a and 6a from the two gene fragments, which indicates that HCV subtype 1b has stronger viability. HCV population genetic characteristics showed HCV-1b, 2a and 6a all experienced a certain degree of population expansion, which is consistent with BSP results, and notably, HCV-6a had a significant expansion in China. Therefore, we suggested that relevant health departments should take more effective measures to control HCV infection, especially strengthening supervision of IDU and publicizing self-protection of risky behaviors of homosexual men, and in addition, it is necessary to organize large-scale molecular epidemiological investigation and to give early warning of HCV outbreak timely.

China Center for Disease Control and Prevention (CCDC) reports that the prevalence of hepatitis C in China has fallen to around 0.4% after the shift to more safe medical practices and illegal commercial blood donations[62]. Therefore, keeping strengthening the management of blood donors and screening of donations is a good way to eliminate HCV-infected people to donate. However, new HCV subtypes or variants will emerge just as HCV-6a has been found in donor group, which may challenge the eligibility of screening strategy and assay. So, continuous surveillance of HCV GT/subtype and population dynamics changing are important to control the risk of transfusion-transmitted HCV in China.

## Supporting information

S1 Table Reference sequences of HCV subtypes used in phylogenetic analysis. (DOCX)

S2 Table Evolutionary rates of HCV subtypes used for Bayesian skyline plot analysis. (DOCX)

S3 Table The geographical distribution of HCV subtypes among blood donors in China. (DOC)
