# Supplementary material for: Hepatitis C virus genotype/subtype distribution and evolution among Chinese blood donors: Revealing recent viral expansion
Source: PLoS One. 2020 Jul 10;15(7):e0235612. doi: 10.1371/journal.pone.0235612 (PMC7351211; doi:10.1371/journal.pone.0235612)
Supplement: S1 Table — (DOCX) [file pone.0235612.s001.docx]

**S1 Table. Reference sequences of HCV subtypes used in phylogenetic analysis.**

| **HCV subtypes** | **GenBank Accession No.** | |
| --- | --- | --- |
|  | **Core** | **E1** |
| 1a | [KT734516 (USA)](https://www.ncbi.nlm.nih.gov/nuccore/KT734516.1), [KF273122 (Malaysia)](https://www.ncbi.nlm.nih.gov/nuccore/KF273122.1), [KT734599 (USA)](https://www.ncbi.nlm.nih.gov/nuccore/KT734599.1),  [KF273120 (Malaysia)](https://www.ncbi.nlm.nih.gov/nuccore/KF273120.1) | [EU781801 (USA)](https://www.ncbi.nlm.nih.gov/nuccore/212675042), [EU781824 (USA)](https://www.ncbi.nlm.nih.gov/nuccore/212675088), [KC844049 (China-Guangdong)](https://www.ncbi.nlm.nih.gov/nuccore/KC844049.1) |
| 1b | [KF273117 (Malaysia)](https://www.ncbi.nlm.nih.gov/nuccore/KF273117.1), [KT734514 (USA)](https://www.ncbi.nlm.nih.gov/nuccore/FJ768835.1), [U01214 (Korea)](https://www.ncbi.nlm.nih.gov/nuccore/U01214.1), [KF273115 (Malaysia)](https://www.ncbi.nlm.nih.gov/nuccore/KF273115.1), [FJ768874 (Vietnam)](https://www.ncbi.nlm.nih.gov/nuccore/FJ768874.1) | [AJ238800 (Germany)](https://www.ncbi.nlm.nih.gov/nuccore/AJ238800.1), [HCU01214 (Korea)](https://www.ncbi.nlm.nih.gov/nuccore/437107), [HQ912959 (China-Shanghai)](https://www.ncbi.nlm.nih.gov/nuccore/HQ912959.1)[,](https://www.ncbi.nlm.nih.gov/nuccore/EU781825.1) [AB249644 (Japan)](https://www.ncbi.nlm.nih.gov/nuccore/AB249644.1), EU781825 (USA) , [HQ912956 (China-Shanghai)](https://www.ncbi.nlm.nih.gov/nuccore/HQ912956.1), [GU451224 (China-Shanghai)](https://www.ncbi.nlm.nih.gov/nuccore/GU451224.1), |
| 2a | [KF676352 (China-Shanghai)](https://www.ncbi.nlm.nih.gov/nuccore/KF676352.1), [AF169004 (Japan)](https://www.ncbi.nlm.nih.gov/nuccore/AF169004.1), [AB690461 (Japan)](https://www.ncbi.nlm.nih.gov/nuccore/AB690461.1), [HQ639943 (China-Liaoning)](https://www.ncbi.nlm.nih.gov/nuccore/HQ639943.1), [AY746460 (Japan)](https://www.ncbi.nlm.nih.gov/nuccore/AY746460.1) | [AF177036 (USA)](https://www.ncbi.nlm.nih.gov/nuccore/AF177036.1), [AF169004 (Japan)](https://www.ncbi.nlm.nih.gov/nuccore/AF169004.1), [AF238485 (Japan)](https://www.ncbi.nlm.nih.gov/nuccore/AF238485.1), [HQ639943 (China-Liaoning)](https://www.ncbi.nlm.nih.gov/nuccore/HQ639943.1), [KC844043 (China-Guangdong)](https://www.ncbi.nlm.nih.gov/nuccore/KC844043.1), [HQ639945 (China-Gansu)](https://www.ncbi.nlm.nih.gov/nuccore/HQ639945.1) |
| 3a | [KF273112 (Malaysia)](https://www.ncbi.nlm.nih.gov/nuccore/KF273112.1), [FJ768836 (Vietnam)](https://www.ncbi.nlm.nih.gov/nuccore/FJ768836.1), [KF057051 (Malaysia)](https://www.ncbi.nlm.nih.gov/nuccore/KF057051.1), [FJ768835 (Vietnam)](https://www.ncbi.nlm.nih.gov/nuccore/FJ768835.1) | [AB792683 (Japan)](https://www.ncbi.nlm.nih.gov/nuccore/AB792683.1), [JQ717260 (India)](https://www.ncbi.nlm.nih.gov/nuccore/JQ717260.1), [KC844041 (China-Guangdong)](https://www.ncbi.nlm.nih.gov/nuccore/KC844041.1), [HQ912953 (China-Shanghai)](https://www.ncbi.nlm.nih.gov/nuccore/HQ912953.1) |
| 3b | [FJ768904 (Vietnam)](https://www.ncbi.nlm.nih.gov/nuccore/FJ768904.1), [FJ768903 (Vietnam)](https://www.ncbi.nlm.nih.gov/nuccore/FJ768903.1), | [KC844044 (USA)](https://www.ncbi.nlm.nih.gov/nuccore/KC844044.1), [JX677149 (China-Guangdong)](https://www.ncbi.nlm.nih.gov/nuccore/JX677149.1) |
| 6a | [FJ768853 (Vietnam)](https://www.ncbi.nlm.nih.gov/nuccore/FJ768853.1), [KJ678763 (China-Guangdong)](https://www.ncbi.nlm.nih.gov/nuccore/KJ678763.1), [EU246930 (Vietnam)](https://www.ncbi.nlm.nih.gov/nuccore/EU246930.1), [KJ678765 (China-Guangdong)](https://www.ncbi.nlm.nih.gov/nuccore/KJ678765.1) | [AY859526 (USA)](https://www.ncbi.nlm.nih.gov/nuccore/AY859526.1), [KJ678765 (China-Guangdong)](https://www.ncbi.nlm.nih.gov/nuccore/KJ678765.1), [KJ678764 (China-Guangdong)](https://www.ncbi.nlm.nih.gov/nuccore/KJ678764.1), [KJ678763 (China-Guangdong)](https://www.ncbi.nlm.nih.gov/nuccore/KJ678763.1), [KJ678754 (China-Guangdong)](https://www.ncbi.nlm.nih.gov/nuccore/KJ678754.1) |
| 6c | [EF424629 (Thailand)](https://www.ncbi.nlm.nih.gov/nuccore/EF424629.1), | [EF424629 (Thailand)](https://www.ncbi.nlm.nih.gov/nuccore/EF424629.1) |
| 6e | [EU246932 (Vietnam)](https://www.ncbi.nlm.nih.gov/nuccore/EU246932.1), [FJ768868 (Vietnam)](https://www.ncbi.nlm.nih.gov/nuccore/FJ768868.1) | [DQ314805 (USA)](https://www.ncbi.nlm.nih.gov/nuccore/DQ314805.1) [JX677023 (China-Guangdong),](https://www.ncbi.nlm.nih.gov/nuccore/JX677023.1) [EU246932 (Vietnam)](https://www.ncbi.nlm.nih.gov/nuccore/EU246932.1) |
| 6f | [DQ835764 (Thailand)](https://www.ncbi.nlm.nih.gov/nuccore/DQ835764.1), [DQ835760 (Thailand)](https://www.ncbi.nlm.nih.gov/nuccore/DQ835760.1) | [DQ835764 (Thailand)](https://www.ncbi.nlm.nih.gov/nuccore/DQ835764.1) |
| 6i | [DQ835770 (Thailand)](https://www.ncbi.nlm.nih.gov/nuccore/DQ835770.1) | [DQ835762 (Thailand)](https://www.ncbi.nlm.nih.gov/nuccore/DQ835762.1) |
| 6j | [DQ835761 (Thailand)](https://www.ncbi.nlm.nih.gov/nuccore/DQ835761.1) | [DQ835761 (Thailand)](https://www.ncbi.nlm.nih.gov/nuccore/DQ835761.1) |
| 6k | [JX183551 (Vietnam)](https://www.ncbi.nlm.nih.gov/nuccore/JX183551.1) | [JX183551 (Thailand)](https://www.ncbi.nlm.nih.gov/nuccore/JX183551.1) |
| 6l | [JX183556 (Vietnam)](https://www.ncbi.nlm.nih.gov/nuccore/JX183556.1) | [JX183556 (Thailand)](https://www.ncbi.nlm.nih.gov/nuccore/JX183556.1) |
| 6m | [DQ835767 (Thailand)](https://www.ncbi.nlm.nih.gov/nuccore/DQ835767.1) | [DQ835767 (Thailand)](https://www.ncbi.nlm.nih.gov/nuccore/DQ835767.1) |
| 6n | [EU246938 (Thailand)](https://www.ncbi.nlm.nih.gov/nuccore/EU246938.1) | [EU246938 (Thailand)](https://www.ncbi.nlm.nih.gov/nuccore/EU246938.1), [EU246937 (Thailand)](https://www.ncbi.nlm.nih.gov/nuccore/EU246937.1) |
| 6o | [EF424627 (Canada)](https://www.ncbi.nlm.nih.gov/nuccore/EF424627.1) | [EU246934 (Vietnam)](https://www.ncbi.nlm.nih.gov/nuccore/EU246934.1) |
| 6v | [EU798761 (China-Yunnan)](https://www.ncbi.nlm.nih.gov/nuccore/EU798761.1) | [EU798761 (China-Yunnan)](https://www.ncbi.nlm.nih.gov/nuccore/EU798761.1), [EU798760 (China-Yunnan)](https://www.ncbi.nlm.nih.gov/nuccore/EU798760.1) |
