# Supplementary material for: Hepatitis C virus genotype/subtype distribution and evolution among Chinese blood donors: Revealing recent viral expansion
Source: PLoS One. 2020 Jul 10;15(7):e0235612. doi: 10.1371/journal.pone.0235612 (PMC7351211; doi:10.1371/journal.pone.0235612)
Supplement: S2 Table — (DOCX) [file pone.0235612.s002.docx]

**S2 Table. Evolutionary rates of HCV subtypes used for Bayesian skyline plot analysis.**

|  | Mean evolutionary rate | Reference |
| --- | --- | --- |
| Core-1b | 5.11E^-4^±7.18E^-6^ | [Yuan, et al. 2013] |
| Core-2a | 6.75e^-4^±1.69e^-4^ |  |
| Core-6a |  |  |
| E1-1b | 1.21e^-3^±2.86e^-5^ | [Lu, et al. 2013] |
| E1-2a | 2.92e^-3^±9.04e^-7^ |  |
| E1-6a | 2.73e^-3^±1.60e^-7^ |  |

Yuan M, Lu T, Li C, Lu L. 2013. The evolutionary rates of HCV estimated with subtype 1a and 1b sequences over the ORF length and in different genomic regions. PLoS One 8(6):e64698.

Lu L, Tong W, Gu L, Li C, Lu T, Tee KK, Chen G. 2013. The current hepatitis C virus prevalence in China may have resulted mainly from an officially encouraged plasma campaign in the 1990s: a coalescence inference with genetic sequences. J Virol 87(22):12041-50.
