# Supplementary material for: Hepatitis C virus genotype/subtype distribution and evolution among Chinese blood donors: Revealing recent viral expansion
Source: PLoS One. 2020 Jul 10;15(7):e0235612. doi: 10.1371/journal.pone.0235612 (PMC7351211; doi:10.1371/journal.pone.0235612)
Supplement: S3 Table — (DOC) [file pone.0235612.s003.doc]

**The geographical distribution of HCV subtypes among blood donors in China**

| **HCV GTs**  **Region** | **1a** | **1b** | **2a** | **3a** | **3b** | **6a** | **6e** | **6n** | **P** |
| --- | --- | --- | --- | --- | --- | --- | --- | --- | --- |
| Luoyang | 0 | 43 | 25 | 0 | 0 | 3 | 1 | 0 | <0.05* |
| Chongqing | 1 | 61 | 22 | 12 | 9 | 25 | 0 | 2 |
| Mianyang | 0 | 63 | 8 | 2 | 4 | 1 | 0 | 0 |
| Urumqi | 0 | 75 | 25 | 5 | 1 | 5 | 0 | 0 |
| Guangxi | 2 | 26 | 2 | 4 | 2 | 22 | 3 | 1 |

***: Monte Carlo Method**

**P<0.05 indicates significant difference**
